# Carers' Medication Administration Errors in the Domiciliary Setting: A Systematic Review

**DOI:** 10.1371/journal.pone.0167204

**Published:** 2016-12-01

**Authors:** Anam Parand, Sara Garfield, Charles Vincent, Bryony Dean Franklin

**Affiliations:** 1 Department of Social Psychology, The London School of Economics, London, United Kingdom / The National Institute for Health Research (NIHR) Imperial Patient Safety Translational Research Centre, Imperial College London, London, United Kingdom; 2 Centre for Medication Safety and Service Quality, Imperial College Healthcare NHS Trust / Research Department of Practice and Policy, UCL School of Pharmacy, London, United Kingdom; 3 Department of Experimental Psychology, University of Oxford, Oxford, United Kingdom; University of Nottingham, UNITED KINGDOM

## Abstract

**Purpose:**

Medications are mostly taken in patients’ own homes, increasingly administered by carers, yet studies of medication safety have been largely conducted in the hospital setting. We aimed to review studies of how carers cause and/or prevent medication administration errors (MAEs) within the patient’s home; to identify types, prevalence and causes of these MAEs and any interventions to prevent them.

**Methods:**

A narrative systematic review of literature published between 1 Jan 1946 and 23 Sep 2013 was carried out across the databases EMBASE, MEDLINE, PSYCHINFO, COCHRANE and CINAHL. Empirical studies were included where carers were responsible for preventing/causing MAEs in the home and standardised tools used for data extraction and quality assessment.

**Results:**

Thirty-six papers met the criteria for narrative review, 33 of which included parents caring for children, two predominantly comprised adult children and spouses caring for older parents/partners, and one focused on paid carers mostly looking after older adults. The carer administration error rate ranged from 1.9 to 33% of medications administered and from 12 to 92.7% of carers administering medication. These included dosage errors, omitted administration, wrong medication and wrong time or route of administration. Contributory factors included individual carer factors (e.g. carer age), environmental factors (e.g. storage), medication factors (e.g. number of medicines), prescription communication factors (e.g. comprehensibility of instructions), psychosocial factors (e.g. carer-to-carer communication), and care-recipient factors (e.g. recipient age). The few interventions effective in preventing MAEs involved carer training and tailored equipment.

**Conclusion:**

This review shows that home medication administration errors made by carers are a potentially serious patient safety issue. Carers made similar errors to those made by professionals in other contexts and a wide variety of contributory factors were identified. The home care setting should be a priority for the development of patient safety interventions.

## Introduction

Medications are mostly taken in patients’ own homes, and yet studies of medication safety have been largely conducted in the hospital setting. Such studies aid hospital staff in better understanding and preventing medication errors[1], but similar research is needed in the community setting.

Literature from Canada and the USA suggest that a significant number of adverse events occur in the home[2–5]. For example, 13% of over 3 million American patients receiving healthcare at home in 2003 suffered an adverse event[5]. This adverse event rate was further supported by findings of 13.2 per 100 Canadian home care cases[6]. A scoping review suggested that adverse drug events (ADE) are the most common adverse events in the home[7]. Although this study did not report how many of these ADEs are caused by errors, indications from elsewhere are concerning, with 30% of 6,718 older home healthcare patients in the US found to have potential medication errors[8]. This is not a problem for the older generation alone; for US children under 6 years old, the average annual rate of medication errors has been reported to be 26.4 per 10,000 population[9]. Within the home setting, the scale of poor adherence to medication regimes by patients themselves is relatively well documented[10, 11]. However, in many cases, a third party is responsible for administering medication at home, and the prevalence and nature of medication errors in the home setting are not widely understood[12].

Within the home, a significant number of people have carers to help with their care[13]. These can be unpaid informal carers such as family members and friends, or paid formal carers such as community nurses and agency carers. Approximately 44 million people provide unpaid care for adult family members and friends in USA[13, 14], there are over 2.7 million unpaid carers in Australia[13, 15], and three in five people in the UK will be carers at some point in their lives[16]. Predictions are that the number of carers will rise by around 60% over the next three decades[16, 17], due to a combination of an increasing older population and higher resource constraints in hospitals. These growing numbers of carers are in a position to hinder or help the safety outcomes of those cared for[18], and medication management is a key part of the daily activities of the home carer. In one study, 78% of 777 informal carers reported that they managed medications as part of their care-giving duties[19].

Previous studies of home medication errors have focused on the healthcare professionals involved and examined prescribing and dispensing errors[20, 21], and little is known about medication administration errors or the role of carers in either causing or preventing them. Some evidence indicates that both informal and formal carers may lack training or knowledge, adding to potential risk in administering medicines[18, 22–24]. While systematic literature reviews have identified issues associated with medication safety in care homes[25], no such systematic review exists for patients’ own homes. We therefore conducted a systematic review of the literature to identify empirical studies that examine how informal and formal carers’ cause and/or prevent medication administration errors (MAEs) within the patient’s home, the types and prevalence of these MAEs, and any interventions to prevent them.

## Methods

### Definitions, Data Sources and Search Strategy

We define a MAE as ‘any deviation between the medication prescribed and that administered’[26, 27]. We take the term ‘prescribed’ to include verbal or written prescriptions and instructions. Our definition of a home carer is any person that provides care and assists in the living activities of patients within their home. This includes both formal paid caregivers working for a healthcare or social service organisation (e.g. agency carers or community nurses) and informal caregivers who are not paid (e.g. relatives or friends of the care recipient).

A review of literature published between 1 Jan 1946 and 23 Sep 2013 was carried out in the online databases EMBASE, MEDLINE, PSYCHINFO, COCHRANE and the Cumulative Index to Nursing and Allied Health Literature (CINAHL). The search strategy involved three conceptual facets: Medication errors/safety, the domiciliary setting, and carer involvement. Care recipients could be adults or children.

To achieve a balance of specificity and sensitivity, multiple iterations and combinations of search terms were tested. Terms were omitted if the sensitivity/specificity were compromised, e.g. the term ‘outpatient’ resulted in a very high number of irrelevant articles. Included terms were based on their use in the literature; e.g. ‘adverse drug event’ is commonly used to include drug errors[7]. The most relevant Medical Subject Heading (MeSH®) term ‘Medication Errors’ was included. For carer involvement, we included healthcare professionals that are most likely to manage medications in the home. S1–S3 Tables present the final search strategies used. The references of the included articles were cross-referenced for missed articles.

### Study Selection

Titles and abstracts were screened against a set of inclusion criteria (S4 Table). Studies selected had to meet all of the following criteria: (a) errors occurred in the home (b) carers were responsible for the delivery of medication and (c) empirical data were provided. We excluded papers describing a single case study, such as an MAE legal case. However, we included papers describing multiple case studies and qualitative studies where there was more than one care-recipient participant. Papers that did not report data for carer-caused MAEs separately to other medication errors or from other administrators (e.g. patients themselves) were excluded, unless over 80% of combined data related to carers, in which case we contacted the author to provide additional details[28–30].

One reviewer (AP) screened titles and abstracts of all articles while a second reviewer (SG) blind-screened a random sample of 10%. Discrepancies were discussed with a third reviewer (BDF) until consensus reached. There was a high percentage of inter-reviewer agreement (90%) between AP and SG and Cohen's kappa suggested good inter-rater reliability (K = 0.73, P = 0.00)[31]. The remaining full text papers were screened by one reviewer (AP), and another randomly assigned 10% was blind-reviewed (SG). There was a good inter-rater reliability (K = 0.95, P = 0.00) between the reviewers, and AP, SG and BDF discussed all articles identified as borderline inclusion/exclusion. All included articles were checked by at least two of the three reviewers to confirm that they met the inclusion criteria. All non-English language papers were reviewed by native language speakers who also all (apart from one) had experience of systematic reviews. If they met the inclusion criteria, the articles were translated by professional translators.

### Data Extraction and Synthesis, and Quality Assessment

Relevant data were extracted from the included articles using a standardised template comprising study details (e.g. study design), medication-specific information (e.g. drug/administration type), outcome information (causative/preventative), sample details (e.g. sample size), and MAE details (e.g. error types). One reviewer (AP) extracted the data and assessed the quality of all articles, while another (SG) extracted and assessed a 10% random sample, with moderate agreement (K = 0.65, P = 0.00).

A quality appraisal tool designed for a range of study designs was used to assess study quality[32]. Each article was scored (on a range 0–2) on each of 10 criteria for qualitative studies, 14 for quantitative studies and all 24 for mixed methods. The scores for each article were added together then divided by the possible total score to give an overall percentage. Additional MAE-specific quality assessment questions were also used, based on similar systematic review assessments[33, 34]. These focused on whether MAEs and their sub-categories were specified/defined, method of MAE detection appropriateness of data collection on MAE causes, and any error validation used.

Since we anticipated that studies would be too heterogeneous to allow for meta-analysis, we used a narrative synthesis to retain original meanings of findings[35]. We inductively identified and grouped contributory factors into a new framework. The review followed PRISMA guidelines[36]. Since we did not conduct a quantitative meta-analysis, we did not formally assess risk of bias but instead described possible sources of bias as part of our quality appraisal of included studies.

## Results

**Fig 1:**
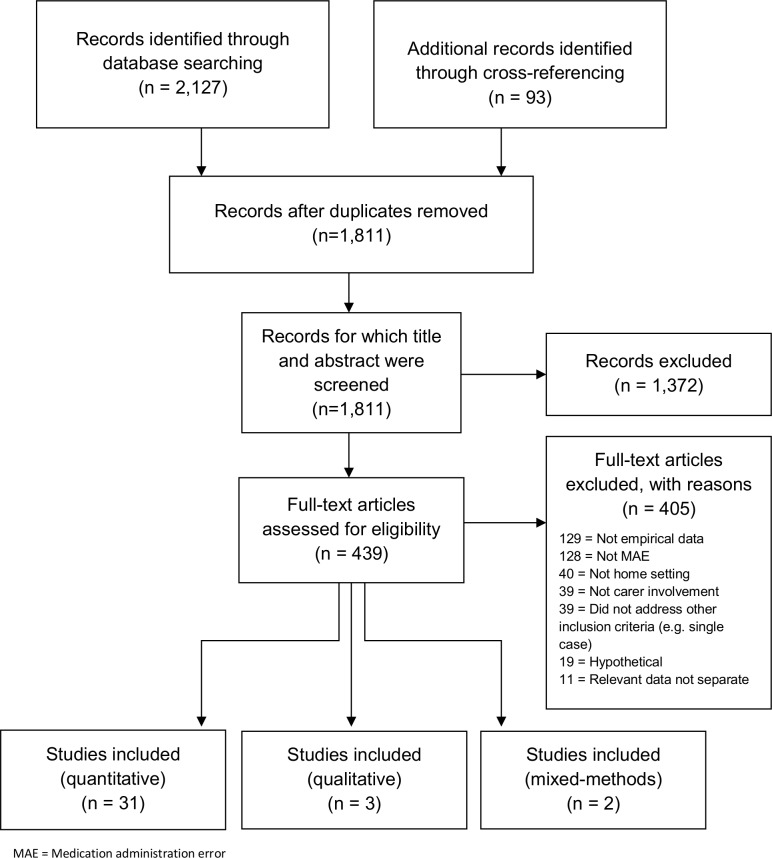
Review stages based on PRISMA Flow Diagram[36]

In total, 1,811 hits remained after duplicates were removed, 439 full text articles were obtained and thirty-six papers met the inclusion criteria. Fig 1 presents the numbers of articles included/excluded at each stage of the review. Study characteristics of the 36 included articles are presented in Table 1, and the total percentage quality assessment scores are in S5 Table.

**Table 1:** Characteristics and summary findings of included studies

First author, Year [Country/Language]	Study Design	Drug /Route of administration	Sample size (n)	Types of carers
**Non-specific medications**				
**Absulem(2011) [US/English][37]**	Quantitative and qualitative cross-sectional convenience sample surveys	Non-specific/non-specific	203 carers	Home health agency nurses
**Azkunaga et al (2013) [Spain/ Spanish][38]**	Quantitative prospective study of charts & surveys	Non-specific/non-specific	400 patients	Parents
**Costa et al [2011] [US/English][39]**	Non-randomised parallel group intervention pilot study. Mixed methods comprising structured closed question interviews and record review.	Non-specific/non-specific	72 patients	Family
**Cohen et al (2008) [US/English][40]**	Quantitative active record review	Non-specific/non-specific	158,520 patients	Parents
**Donelan et al [2002] [US/English][18]**	Quantitative cross sectional surveys	Non-specific/non-specific	1,002 carers	Family or friends
**Jonville et al (1991) [France/English][41]**	Quantitative prospective cross sectional surveys	Non-specific/rectal, oral, cutaneous, eye, ear, nose and throat, parenteral	896 carers	Family & babysitter
**Kaushal et al (2007) [US/English][42]**	Quantitative prospective cohort study, mixed methods of duplicate prescription review, telephone surveys and chart review.	Non-specific/non-specific	1715 carers	Parents, guardians, and other home carers
**Lemer et al (2009) [US/English][43]**	Quantitative mixed methods prospective cohort study with structured closed question interviews, a review of duplicate prescriptions and chart review.	Non-specific/non-specific	1685 paediatric patients	Parents
**Lifshitz & Gavrilov (2000) [Israel/English][44]**	Quantitative retrospective record review	Non-specific/non-specific	1,143 cases	Family
**Llewellyn et al (2003) [Australia/English][45]**	Quantitative randomized controlled trial intervention and questionnaire	Non-specific/non-specific	45 carers	Parents
**Ni et al (2013) [China/Mandarin][46]**	Quantitative convenience sample surveys	Non-specific/non-specific	332 carers	Parents
**Pelissier-delour et al (2007) [France/French][47]**	Quantitative surveys	Non-specific/non-specific	64 patients	Parents
**Ranelli & Hansen (1997) [US/English][48]**	Qualitative interviews	Non-specific/non-specific	29 carers	Family
**Schaefer et al (2008) [US/English][49]**	Quantitative retrospective review of national records	Non-specific/non-specific	63 hospitals	Parents
**Slatter et al (2004) [UK/English][50]**	Qualitative interviews	Non-specific/non-specific	17 carers	Parents
**Taylor D (2009) [Australia/English][30]**	Quantitative prospective mixed methods observational study, record review and surveys	Non-specific/non-specific	491 cases	Family & babysitter
**Travis et al (2000) [US/English][51]**	Qualitative interviews	Non-specific/non-specific	23 carers	Family
**Walsh et al (2011) [US/English][29]**	Quantitative mixed method study with observations, medication review and event classification	Non-specific/non-specific	56 carers	Parents or guardians
**Walsh et al (2013) [US/English][28]**	Quantitative mixed method study with observations, medication review and event classification	Non-specific/non-specific	92 patients	Parents or guardians
**Zandieh et al (2008) [US/English][52]**	Quantitative mixed method prospective cohort study surveys and record review	Non-specific/non-specific	55 children	Parents or guardians
**Conroy et al[2003] [UK/English][53]**	Quantitative cross sectional surveys	Various over-the-counter medicines/Oral	424 carers	Family
**Antipyretic-analgesic medications**				
**Alander(2000) [US/English][54]**	Quantitative retrospective record review	Paracetamol/oral and rectal	322 patients	Parents
**Alomar et al (2011) [Saudi Arabia/English][55]**	Prospective cross-sectional structured closed question interviews and partial observations	Paracetamol/oral and rectal	178 carers	Carers of children
**Goldman & Scolnik [2004] [Canada/English][56]**	Cross-sectional structured closed question interviews	Paracetamol/not specified	213 carers	Parents
**Gribetz & Cronley [1987] [US/English][57]**	Cross-sectional structured closed question interviews	Paracetamol/not specified	88 carers	Parents
**Guberman [1990] [Israel/Hebrew][58]**	Prospective structured closed question interviews	Paracetamol/mostly oral	101 carers	Parents
**Heubi et al (1998) [US/English][59]**	Retrospective case series record review	Paracetamol/not specified	47 patients	Parents
**Hyam et al (1989) [Israel/English][60]**	Cross-sectional structured closed question interviews	Paracetamol/not specified	100 patients	Parents
**Li et al (2000) [US/English][61]**	Quantitative cross-sectional study with questionnaires	Paracetamol & ibuprofen/ not specific	200 patients	Family
**McErlean et al (2001) [US/English][62]**	Quantitative prospective convenience sample structured closed question interviews	Antipyretic drugs—paracetamol & ibuprofen/non-specific	138 patients	Family
**Rivera-penera et al (1997) [US/English][63]**	Quantitative retrospective case series record review	Paracetamol/non-specific	73 records of patients	Parents
**Other Specific medications**				
**Mattar et al (1975) [US/English][64]**	Quantitative mixed methods structured closed question interviews and quantitative observations	Medication for acute otitis media/oral	300 patients	Parents
**McMahon et al (1997) [US/English][65]**	Quantitative intervention randomised convenience sample, mixed methods with quantitative observations	Antibiotic suspension/Oral	90 carers	Parents
**Moretti et al (2013) [France/French][66]**	Quantitative mixed methods retrospective case series record review and structured closed question interviews	Diazepam/Included intrarectal	114 patients	Parents
**Ryu & Lee (2012) [South Korea/English][67]**	Quantitative observations	Coben [chlorphenamine and phenylephrine] syrup/Oral	282 carers	Family
**Taylor J. et al (2006) [US/English][68]**	Quantitative prospective case series mixed methods with quantitative observations and record review	Chemotherapeutic medications)/Oral	69 patients	Parents or guardians

### Study Samples, Methods and Settings

The majority of studies were carried out in the US[18, 28, 29, 37, 39, 40, 42, 43, 48, 49, 51, 52, 54, 57, 59, 61–65, 68]. The remainder were from Saudi Arabia[55], Spain[38], UK[50, 53], Canada[56], Israel[44, 58, 60], France[41, 47, 66], Australia[30, 45], China[46] and South Korea[67]. Only one studied formal carers, i.e. home health agency nurses[37]; all others investigated informal carers. Of the 35 that included informal carers, 15 included parents among other carers[18, 28–30, 39, 41, 42, 44, 52, 53, 55, 61, 62, 67, 68] and 18 studied only parents[38, 40, 43, 45–47, 49, 50, 54, 56–60, 63–66]. Moreover, the parent samples were mostly mothers, for example with over 90% mothers in 7 studies[28, 29, 53, 57, 61, 64, 65] and 78.3% in another[67]. The remaining two studies focused on older adult care-recipients who were mostly cared for by their adult children carers or by their spouses[48, 51]. Although many studies did not explicitly exclude informal carers who were friends of the care-recipient, and included them in their definition of caregiver[18], no studies focused solely or significantly on this type of carer.

The definition of a MAE was often not reported; in some cases this was because the study focus was wider and included any type of medication error. Those that defined a MAE classified it as deviation from prescriptions and/or instructions/accepted guidelines. For those investigating paracetamol dosing errors, a tolerance of 10-15mg per dose was most commonly used.

The majority of studies (28/36) were quantitative studies that used retrospective record review, questionnaires or structured closed question interviews, quantitative observations, or a combination of these. Three studies reported interventions[39, 45, 65], three were qualitative[48, 50, 51], and two used mixed-methods (qualitative and quantitative)[18, 37]. Most (25/36) studies included self-reports, either alone (15 studies) or together with other data collection methods (10 studies). The remaining studies did not use self-report and instead used more objective data collection methods, such as record review and observations.

Quality assessment scores ranged between 50–100%, with most (26/36) scoring above 80%. Common methodological issues were undefined error categories and convenience sampling. In some cases, validation of errors would have strengthened the study and those that used retrospective study or self-reported errors as their main outcome measure may suffer from additional bias.

While all studies focused on MAEs in the home, some (24) of the data collection took place elsewhere: in hospital (5)[39, 51, 54, 64, 68], specifically in emergency departments (9) [38, 40, 49, 55–57, 61, 62, 66], as well as medical centres (4)[44, 58, 63, 65], outpatient clinics (2)[29, 42], a home healthcare agency[37], a poisons centre[30] and other clinics/practices (2)[28, 43].

### Types and Prevalence of MAE

The most common type of MAE was dosage errors, [28–30, 38–41, 44, 46, 47, 49, 51, 53–63, 65–68]. Omitted administration[28, 29, 37, 39, 50, 51, 66, 68] and wrong medication[30, 37, 43, 44, 46, 49, 61] were also common. Other types of MAEs were wrong time of administration[30, 46], wrong route of administration[30, 41, 49] wrong patient[41], inappropriate combination with other drugs[47], wrong preparation technique[37, 47], giving expired medication[29, 49], wrong formulation[41, 49], age-inappropriate drugs[41]; not washing equipment[47] and either not completing treatment[42, 43, 52] or not stopping treatment[48].

It is difficult to compare error rates across studies due to the differing methods used to identify errors. For errors not related to a specific medication group, the carer administration error rate ranged from 1.9 to 33% of medications administered[29, 42, 47, 52], 12% of carers administering medication[18], and 70% of patients receiving medication[28]. In addition, 7.8% (26) of carers reported that they had given an insufficient dose, 6.6% (22) reported that they had given an overdose and 5.4% (18) reported that they had given the wrong medicine[46]. A separate study evaluated the relative percentage of administration versus non-administration errors and showed 1.7% of medication errors and 22% of the near misses occurred at the administration stage[43].

A small number of studies endeavoured to assess the impact of medication errors. Three studies used clinical record review to determine the percentage of cases of poisoning caused by medication errors. One study found that 10% of cases of medication poisoning in children under seven were caused by dosage errors, although it is unclear whether all occurred at administration[38]. Another study found that in 15.9% cases of acute poisoning, including medication and other agents, there was an error in dosage of a medication and in 8.8% cases an incorrect drug was given by a member of the child's family[44]. A similar study found that therapeutic errors in children (<16yrs) reported to a poison centre were mostly administration errors by carers (80.9%, 397 out of 491), rather than the patients themselves[30]. A fourth study found only 1.6% of unintentional overdoses in 1–4 year olds had documentation of dosing errors on the part of the caregiver[40].

#### Studies of specific types of medication

Sixteen studies set out to examine specific types of medication and some focused on specific routes of administration: oral, or a combination of these including rectal routes[41, 54, 55, 58]. Antipyretic-analgesic medications were the most common group of medications studied. Six studies determining the accuracy of parents administering paracetamol to their children found that either over 50% of parents gave, or over 50% children received, an incorrect dosage[55–58, 61, 62]. Frequency of paracetamol dosing was prone to some error, e.g. almost a quarter of parents gave paracetamol or ibuprofen less frequently than recommended or gave Ibuprofen more frequently than recommended[53, 61].

Almost all (92.7%) parents of children prescribed antibiotics for acute otitis media reported giving fewer than the prescribed number of days of antibiotics[64]. In the home management of children with epilepsy, diazepam was not administered by parents in 61% (20 out of 33) of cases where it should have been according to treatment guidelines[66]. Interviewed parents or guardians administering chemotherapeutic medications had an administration error rate of 7% of medicines[68]. A clinical record review found that 8% of visits to an emergency department for cough/colds were caused by medication errors (including MAEs)[49].

### Causes of MAEs and Potential Contributory Factors

There were recurring factors cited across the studies, Table 2 presents the findings on how these affected MAEs. The most common risk factors identified were equipment issues and miscommunication or poor understanding of illness, instructions or calculations. Polypharmacy, storage, type of medication, carer education level, language, and dosage change were identified as factors across several studies. The factor with the most contradictory evidence was age of children care-recipient, with opposing results on whether older or younger children are at more risk of MAEs. The only other factor for which not all studies showed consistent findings was healthcare professional communication and carers’ understanding of instructions. Although nine of 12 studies found this to be significantly associated with MAEs, the others did not.

**Table 2:** Causes and potential contributory factors of informal carers’ MAEs

Contributory Factor	Contributory Sub-Factor	Evidence
INDIVIDUAL CARE RECIPIENT FACTOR	Age of child	• Being a care-recipient child below the age of 5 years was found to be a significant predictor of an increased risk for a MAE[43]. • Infants were found to be significantly more likely to receive an incorrect dose of medication than older children[61]. • Underdosing was most commonly noted in both younger and lighter children. The mean age and weight of the children were significantly less in the underdosed group and overdosed group compared with the appropriate dose group[57]. • Surpassing the recommended maximum number of doses was more likely with increasing age of the child care-recipient[53]. • From the age of 12 months, administration of the recommended dose declined with the increasing age of the child (regardless of an increase in dosage given): 1-2yrs (81%), 2-3yrs (65%), 4-6yrs (55%), 6-8yrs (43%). Of those between the age of 4–11 months 62% gave a recommended dose[60] • Children aged between 1–4 most frequently had unintentional overdoses and 10 times the rate in other (older and younger) age groups (3.2 versus 0.3 ADEs per 1000 persons)[40]. • 71% (10 of 14) children under 10 years old (all receiving multiple overdoses by parents) had a severe toxic condition of the liver, compared with 31% (18 of 59) in the older group[63].
INDIVIDUAL CARER FACTOR	Age of carer	• More medication errors were reported by older carers (>65yrs), despite fewer older caregivers in the study sample[18].
	Educational level of carer	• Carers with less education reported more MAEs[18]. • Carers with a low educational level (below 12^th^ grade–school aged around 17–18) had complied less with medication prescriptions[64].
	Carer’s time and other responsibilities	• Carers who continued to work or who had other family/caregiving responsibilities reported more missed doses, regardless of their administration schedules[51]. • Following a treatment regimen in between everyday activities or special occasions was raised as a potential contributor to missing administration[50].
	Language of Carer	In English speaking countries: • Non-English speaking parents gave the recommended dose of paracetamol less frequently than English-speaking parents[56]. • Accuracy of dosage differed across language groups, with Spanish speakers less accurate in dosing than English speakers, however this was found to be not significant[65]. • Bottle labels in English contributed to MAEs for non-English speakers, despite a consultation in the mother tongue of the carer[29].
	Health of carer	• Carers with poorer health reported more MAEs than those with better health[18].
	Carer marital status	• Single mothers had lower compliance with prescription administration compared with married mothers (p = .004)[64].
MEDICATION FACTORS	Polypharmacy	• Children with multiple prescriptions were at a significant increased risk of having a preventable ADE[52]. • Medication errors increased with the number of administered medications[18]. • Taking more than one medication increased the risk of a MAE (odds ratio: 1.60, 95% confidence interval)[43]. • 20% of parents were reluctant to give more than one medication at a time[64].
	Type of medication	• The most common drugs involved in preventable ADEs in paediatric outpatients were amoxicillin/amoxicillin-clavulanate, inhaled steroids, topical anti-fungals, antihistamines, and inhaled bronchodilators [52]. • More cough and cold medication-related emergency visits involved medication errors (e.g. administering an overdose) than visits from all other medications combined[49]. • MAEs were most prevalent, in nebulised therapy, followed by oral antibiotics [50]. • More errors were found with non-chemotherapy medications rather than chemotherapy medication[28].
	Route of administration	• Paracetamol given via the rectal route of administration had a significantly greater rate of supratherapeutic doses than oral administration (9/28 [32%] versus 39/149 [26%]), respectively (95% CI = 0.14 to 0.48)[55]
	Medication supply	• 25% of 20 parents who did not administer diazepam did not have any diazepam (25%)[66]. • Parents raised the issue of being given incorrect products[50]. • Carers created complex strategies to maintain their supply when faced with a host of different sources (e.g. pharmacies, samples and mail order), various reimbursement sources and variable doses[51]. • A double dose of senna was identified due to two filled prescription bottles.[39] • Not replacing spilled medication or broken medication bottles that resulted from difficulty in administering the medication [64].
ENVIRONMENTAL FACTORS	Storage	• Families that reported MAEs said they had not stored the products in their original container[38]. • Inappropriate places of storage included under the sink, in the refrigerator or bathroom. Few stated they stored them in a locked cupboard[53].
	Equipment	• Use of inappropriate measuring equipment was often identified as potential contributory factors[53, 62], particularly the use of teaspoons. • A 100% dosage accuracy was found in the group that received their prescription along with a syringe that had a line marked at the correct dose, compared to 37% correct dosage accuracy from a group that only received a prescription and verbal instruction. 83% accuracy in dose was found when a group with provided with the syringe, prescriptions and demonstration alone[65]. • Percentage of incorrect use for the following measuring aids were: Dosing spoons 44% (used by 20% of carers), teaspoons 100% (used by 17%), syringes 60% (used by 17%), and droppers 100% (used by 10%)[65]. • The mean dose given with an infant dropper was lower (6.4 mg/kg per dose, p < 0.0002) than the mean doses given with other measuring devices[57]. • Parents reported difficulties in using IV lines and nebulizers/inhalers, and the supply of incorrect equipment[50]. • Parents sometimes deviated their administration technique from the doctor’s instructions because they did not have the proper equipment[29]. • 3.6% (12) carers stated that they did not know how to open child safe containers[46].
PRESCRIPTION COMMUNICATION FACTORS	Communication with healthcare professionals & carers’ understanding of instructions or medication/illness	• In a case study example, lisinopril and amlodipine were prescribed at discharge; however, the daughter did not understand that the mother was to take the drugs[39]. • Of 20 parents who did not administer diazepam when they should, 60% reported it was due to the complicated administration information, 35% said they were unaware, and 2% potentially had misinformation on dosage[66]. • Inadequate and erroneous understanding by parents about their children’s medications/illness was identified, however no significant relationship was found between this understanding and MAEs[64]. • Advice to parents on administration was not associated with MAEs[43]. • The source of information on medication amount was not significantly different between the correct group and the incorrect group[61]. • Parental MAEs resulted from misunderstanding instructions, disregarding medication labels or following them rather than other given instructions[28] • Carers who said that medication dose is based on weight were significantly less likely to give an incorrect dose of medication (RR 0.71, p = 0.03)[61]. • The mean administered dose of paracetamol was 62% of that recommended when the calculation was made based on the care-recipient’s age, and 64% when it was calculated by body weight[60]. • Miscalculation of dose was found to be a factor in wrong dose errors[59]. • Three causes of MAEs were due to a new carer being unfamiliar with medications[30] • More errors occurerd where medication labels did not specify dosage but directed carers to consult a healthcare professional[49]. • 25.3% (84) of carers did not know how to administer medicine to their child, 7.2% (24) forgot what their pharmacist or doctor tole them to take note of at administration and 5.4% (18) reported that they dound it difficult to understand medicine information pamphlets[46].
	Dosage change	• Becoming accustomed to a medication and consequently not reading new instruction labels was identified[51]. • Administration errors were most often caused by confusion regarding a change in dose of a medication[29]. • Unnoticed expired medicines used by parents were usually PRN (taken as needed)[29].
PSYCHOSOCIAL FACTORS	Panic / Cognitive failure	• 20% of 20 parents who did not administer diazepam when they should have, reported that they panicked at the time it was needed, while 15% said it did not occur to them[66].
	Fear of spillage	• Mothers reported not filling the entire spoon due to fear of spilling the medication, particularly when dosage was half or three quarters of a spoonful rather than one whole spoonful[64].
	Carer-to-carer communication	• In 89 (18.1%) cases a lack of communication between two carers resulted in both giving a dose to the child. In 80 of these cases, the carers were both parents. Poor communication between parents was reported 97 times, 10 times between parents and grandparents, and 4 times between other carers[30].

**MAE(s)** = Medication Administration Error(s)**ADE(s)** = Adverse Drug Event(s)

### Interventions and Carers’ Activities to Prevent MAEs

Preventative activities by carers included planning medication schedules around meal/bedtime routines for caregivers with predictable schedules[51] and using a multi-compartment compliance aid[39]. Carers identified the need to be constantly vigilant in monitoring care-recipients’ conditions[51], and often detected errors; in one study 85% of MAEs were detected by a family member (compared with 12% by healthcare professionals)[41]. However, in 16% of 152 ADE cases, parents did not disclose their errors[42].

Only three intervention studies identified strategies to prevent carers’ MAEs. One was a randomised convenience sample of parents administering antibiotics that tested instructions, plus administration demonstration and (marked/non-marked) equipment for medication administration[65]. The medication administration demonstration along with a marked syringe at the correct dosage level improved the accuracy of dosage (100% accuracy) compared with prescriptions, syringe and demonstration alone (83% accuracy) or compared with prescription alongside verbal instructions alone (37% dose accuracy)[65]. The second study was a randomised controlled trial of 10 weekly lessons carried out in the parent’s home on child health and home safety[45]. The lessons significantly increased the parents’ health behaviours on how to use medicines alongside a significant increase in parent knowledge and skills in using medicines safely (compared with visits and current services alone)[45]. The third study implemented a hospital-to-home transitional care nurse coaching intervention for people managing complex medication regimens at home. The intervention comprised of interviews and medication reconciliation at the hospital and following hospital discharge, followed by a home visit to observe medication use. The study identified that 62.5% (10 out of 16 home visits) more discrepancies between medications prescribed and administered were detected at home visits than by telephone, and that coaching the caregiver responsible for the medication regimen was important in resolving such discrepancies[39].

## Discussion

This review has shown that medication errors made by carers in patients’ homes are a potentially major patient safety issue. Carers appear to make the full range of errors made by professionals in other contexts and there are a wide variety of causes and contributory factors. Very few interventions have been trialled to support carers and reduce MAEs in the home.

Prevalence rates of carer MAEs varied. Elsewhere, general medication error rates for young children were identified to be 26.42 per 10,000 population[9]. Other research further supports the findings that home MAEs are a significant issue. For example, of 2,348 medication errors reported to the National Poisons Information Centre in Ireland, 2,135 were caused by errors in the home setting, the majority of which were due to administration errors[69].

MAEs caused by carers were wide-ranging, however, in many studies, these errors were highlighted as examples of MAEs identified rather presenting a comprehensive list. Reviews on medication errors in the home support the identified administration errors, particularly wrong dosage[7, 70].

It is likely that many of the identified contributory factors are interlinked, e.g. understanding instructions could be linked with carers’ language and education. The challenge is how to address vulnerable carer groups, particularly as research may not be targeting the groups most in need[71]. Recommendations include tailoring interventions to different carer groups[71], however this review identified a very limited number of interventions.

Many of the identified factors and solutions are supported by research beyond this review, such as on equipment[72, 73]. Literature reviews on home medication errors with older adults advocate medicine reconciliation and better communication with healthcare staff in order to address polypharmacy and medicine-related factors[74, 75]. The agreement with our findings on parental administration indicate overlapping risks in caring for old and young with medication. It also supports an emerging case for better communication between carers and health professionals, reinforced by hypothetical scenarios of medication administration by carers showing their increased adherence to instructions when doctors provided explanations[76].

### Review and Research Strengths and Limitations

This is the first review to explore informal and formal carers’ MAEs at home. A strength is that there were no restrictions on article language, allowing for broad inclusion of the international literature. Additionally, the reviewers held a relevant mix of expertise on patient and medication-related safety, caregivers, and homecare. While the included studies were heterogeneous and did not allow for substantial quantitative comparison, there were clearly identifiable groupings of error types, prevalence and causes as well as clear research gaps that emerged from the narrative nature of the review.

Prevention of MAE was less easily identifiable than error causation, due to the potentially more ambiguous and broader range of preventative activities. This difficulty may have compromised the extent to which we identified evidence on carers preventing errors. The quality scores are subjective and limited by the quality assessment scale used and its restricted criteria. A major limitation was that the systematic review was only up to September 2013.

This review emphasises the need for more research on home care medication safety, particularly as medication-related models from the hospital setting have been deemed unsuitable for application in the home[77]. Our review has additionally discovered the gaps within the current literature. Most studies were based in the USA with mother carers, despite statistics showing that there are only 30% more females than males in caring roles in the USA[78]. More research is required on formal carers who frequently administer medications in the home, and informal carers who are friends, spouses or adult children of the care-recipient. Currently, we know very little about these carers or their medication-related activities and any additional risk factors. For instance, some carers may make more errors due to sensory deficits or cognitive problems[79, 80]. More research with formal caregivers would allow for a useful comparison and distinction between formal and informal carers, particularly in regards to potential differences in causes of MAEs and their implications for tailored interventions. Further investigation is also required to clarify whether younger or older children are at higher risk of MAEs and whether this is context-dependent. Studies would additionally benefit from random sampling strategies and validation of errors. The latter, in particular, would help minimise subjective biases resulting from self-reports, especially as parents do not always disclose their MAEs[42]. Despite not restricting the articles by the English language, there is not a full representation of countries researching this topic. Other countries may have their own specific set of barriers contributing to errors, such as poor access to up-to-date medicine information[81]. Preventative interventions are presently scarce.

The data collected from home and clinical settings were analysed together. However, observed demonstrations of administration in clinical settings may be different from the home setting, for example, there may be more distractions in either the home or clinical environment and equipment might have been available in clinics that is not available in the home. As found in an earlier systematic review on MAEs in hospitals[82], the MAE definitions and error categories are rarely reported, which hinders study comparison.

### Implications for Clinical Practice

Specific actions for clinical practice could include interventions to improve communication between healthcare professionals and carers. This would involve consideration of carers’ understanding of the information, particularly on the prescription/medication labels, administration techniques, and on what to consider if the prescription changes, and the importance of storage. Carers could be encouraged to keep notes of this guidance to aid recall of instructions. Similarly, more detailed advice can be provided to carers on how they communicate with one another (e.g. inter-parental communication). This could also form part of more formal carer training and home visits would allow for more objective verification of appropriate administration. Carer calculations can be checked for accuracy, including those dependent on the weight of the care-recipient. Similarly, appropriate and inappropriate equipment (e.g. teaspoons) should be identified. Where possible, tailored equipment (e.g. with marked dosage lines) could be provided. Such consultations and home visits could further incorporate more questions regarding carer concerns (on medication management) and their mental well-being.

These actions should be considered particularly when the care-recipient is taking multiple medications or when there is likelihood of frequent dosage changes. Those administering ‘taken as needed’ medications should be particularly made aware of the importance of expiry dates. Extra care could also be taken for more complicated routes of administration and potentially with certain types of medication that may be more susceptible to error (e.g. cold/cough medicines), as well as for informal carers who are older, single, have lower educational levels, poorer health, or are non-native speakers.

## Conclusion

This review reinforces the need to consider potential medication safety implications in home healthcare[83]. The evidence reveals carer administration error rates ranging from 1.9 to 33% of medications administered, 12 to 92.7% of carers administering medication, and 70% of patients receiving medication. Contributory factors include individual carer, environmental, medication, prescription communication, psychosocial factors and care-recipient factors. Useful interventions to prevent MAEs include carer training, tailored equipment, and home medication checks. There is a strong argument for training carers and communication between carers and healthcare professionals, as well as attention required to medication supplies, medication equipment used, and type and number of medications administered. The findings support calls for authorities to consider the safety standards and social policy required in homecare[18, 84]. The review additionally outlines the gaps in the research around non-parental carers and formal carers and the need for further studies of interventions. We feel it is important to tackle the issues highlighted by this review as the numbers of carers around the world grow, and with them the prevalence of medication administration.

## Supporting Information

S1 Table Search strategy formula for MEDLINE & EMBASE & PSYCHINFO databases via OvidSP (DOCX)

S2 Table Search strategy formula for CINAHL database (DOCX)

S3 Table Search strategy formula for COCHRANE database (DOCX)

S4 Table Inclusion/Exclusion Criteria (DOCX)

S5 Table Quality Assessment Scores (DOCX)
