# Supplementary material for: Carers' Medication Administration Errors in the Domiciliary Setting: A Systematic Review
Source: PLoS One. 2016 Dec 1;11(12):e0167204. doi: 10.1371/journal.pone.0167204 (PMC5132322; doi:10.1371/journal.pone.0167204)
Supplement: S1 Table — (DOCX) [file pone.0167204.s001.docx]

**S1 Table - Search strategy formula for MEDLINE & EMBASE & PSYCHINFO databases via OvidSP**

| **Facets** | **Steps** | **Search strategy formula for MEDLINE & EMBASE & PSYCHINFO** |
| --- | --- | --- |
| Medication error/Safety | 1 | Medication Errors/ or (medication* error* or medication* related error* or drug* error* or drug* related error* or medication* mistake* or medication* related mistake* or drug* mistake* or drug* related mistake* or adverse drug* event* or administ* error* or medicine* error* or medicine* related error* or dos* error* or medication* management or drug* management or medication* safe* or safe medication* or medicine* management or medicine* safe* or safe medicine* or manag* medication* or manag* medicine* or manag* drug* or management of medication* or management of medicine* or management of drug* or medication* administ* or medicine* administ* or drug administ* or adminst* of medication* or adminst* of medicine* or adminst* of drug*).ti,ab. |
| Home setting | 2 | (residential or residence or retirement or long term care facilit* or home* or domicil* or community or social care or hous* or assisted living).ti,ab. |
| Carer involvement | 3 | (carer* or caregiver* or care giver* or care aid* or family or relatives or nurse* or matron* or occupational therapist* or midwi* or parent*).ti,ab. |
|  | 4 | 1 and 2 and 3 |
|  | 5 | remove duplicates from 4 |

* = Truncation (e.g. administ* picks up administration or administering

/ = Subject heading term *(the MeSH term was not available in PSYCHINFO)*
