# Supplementary material for: Carers' Medication Administration Errors in the Domiciliary Setting: A Systematic Review
Source: PLoS One. 2016 Dec 1;11(12):e0167204. doi: 10.1371/journal.pone.0167204 (PMC5132322; doi:10.1371/journal.pone.0167204)
Supplement: S2 Table — (DOCX) [file pone.0167204.s002.docx]

**S2 Table - Search strategy formula for CINAHL database**

| **Facets** | **Steps** | **Search strategy formula for CINAHL** |
| --- | --- | --- |
| **Medication Error MeSH term** | 1 | (MM "Medication Errors") |
| **Medication error/Safety** | 2 | (medication* error* or medication* related error* or drug* error* or drug* related error* or medication* mistake* or medication* related mistake* or drug* mistake* or drug* related mistake* or adverse drug* event* or administ* error* or medicine* error* or medicine* related error* or dos* error* or medication* management or drug* management or medication* safe* or safe medication* or medicine* management or medicine* safe* or safe medicine* or manag* medication* or manag* medicine* or manag* drug* or management of medication* or management of medicine* or management of drug* or medication* administ* or medicine* administ* or drug administ* or adminst* of medication* or adminst* of medicine* or adminst* of drug*) |
| **Home setting** | 3 | (residential or residence or retirement or long term care facilit* or home* or domicil* or community or social care or hous* or assisted living) |
| **Carer involvement** | 4 | (carer* or caregiver* or care giver* or care aid* or family or relatives or nurse* or matron* or occupational therapist* or midwi* or parent*) |
|  | 5 | 1 or 2 |
|  | 6 | 3 and 4 and 5 |

* = Truncation (e.g. administ* picks up administration or administering

/ = Subject heading term
