# Supplementary material for: Carers' Medication Administration Errors in the Domiciliary Setting: A Systematic Review
Source: PLoS One. 2016 Dec 1;11(12):e0167204. doi: 10.1371/journal.pone.0167204 (PMC5132322; doi:10.1371/journal.pone.0167204)
Supplement: S3 Table — (DOCX) [file pone.0167204.s003.docx]

**S3 Table - Search strategy formula for COCHRANE database**

| **Facets** | **Steps** | **Search strategy formula for COCHRANE** |
| --- | --- | --- |
| **Medication Error MeSH term** | 1 | MeSH descriptor: [Medication Errors] this term only |
| **Medication error/Safety** | 2 | ("medication* error*" or "medication* related error*" or "drug* error*" or "drug* related error*" or "medication* mistake*" or "medication* related mistake*" or "drug* mistake*" or "drug* related mistake*" or "adverse drug* event*" or "administ* error*" or "medicine* error*" or "medicine* related error*" or "dos* error*" or "medication* management" or "drug* management" or "medication* safe*" or "safe medication*" or "medicine* management" or "medicine* safe*" or "safe medicine*" or "manag* medication*" or "manag* medicine*" or "manag* drug*" or "management of medication*" or "management of medicine*" or "management of drug*" or "medication* administ*" or "medicine* administ*" or "drug administ*" or "adminst* of medication*" or "adminst* of medicine*" or "adminst* of drug*"):ti,ab |
| **Home setting** | 3 | (residential or residence or retirement or "long term care facilit*" or home* or domicil* or community or "social care" or hous* or "assisted living"):ti,ab |
| **Carer involvement** | 4 | (carer* or caregiver* or care giver* or care aid* or family or relatives or nurse* or matron* or "occupational therapist*" or midwi* or parent*):ti,ab |
|  | 5 | #1 or #2 |
|  | 6 | #3 and #4 and #5 |

* = Truncation (e.g. administ* picks up administration or administering

/ = Subject heading term
