# Supplementary material for: Carers' Medication Administration Errors in the Domiciliary Setting: A Systematic Review
Source: PLoS One. 2016 Dec 1;11(12):e0167204. doi: 10.1371/journal.pone.0167204 (PMC5132322; doi:10.1371/journal.pone.0167204)
Supplement: S4 Table — (DOCX) [file pone.0167204.s004.docx]

**S4 Table - Inclusion/Exclusion Criteria**

|  | Type of paper | Country | Language | Study design | Setting | Population | Outcome |
| --- | --- | --- | --- | --- | --- | --- | --- |
| Inclusion  criteria | -Empirical data  -Case studies | Any | Any | Any  (e.g. qualitative, quantitative or mixed methods) | Individuals’ homes or residences that house people but that do not have 24 hour provision of care  (e.g. a patient’s house, sheltered housing) | -Carers. Defined as ‘any person that provides care for and assists with the living of patients within the patient’s home’.  -Includes formal and informal carers (e.g. district nurses, community matrons, and family members of the patient) | -Medication administration errors (MAEs) caused or prevented by carers  -Where MAEs are present, we include the causative or preventative factors of MAEs by carers  -A MAE is defined as ‘any deviation between the medication as prescribed *or instructed* and that administered’ (Barber et al, 2009)’ We have added the words “or instructed” into this definition to include administration deviating from bottle labels, doctor verbal instructions, accompanying patient held information etc  -This includes MAEs that result due to dispensing errors  -We include MAEs that result due to over the counter (OTC) errors  -Reported MAE is included as long as it refers to actual (not hypothetical) MAE.  -We include studies if over 80% of the data presented were carer MAEs. |
| Exclusion  criteria | -Commentary papers  -Editorials  -Books  -Leaflets  -Meeting notes  -Literature reviews  -Single person case studies (i.e. articles on 1 or 2 single cases, rather than a study with multiple subjects)  -Conference abstracts  -Dissertations | None | None | None | Any homes that has 24 hour provision of care  (e.g. care/nursing homes) | -Healthcare professionals that do not assist with living in the home or who visit the patient’s home only to administer medication.  (e.g. GPs or community pharmacists). -Carers that do not assist with medication  (e.g. social workers) | -Other types of medication errors (e.g. prescribing or dispensing errors)  -MAEs that are not caused or prevented by carers  -Nutrition/dietary enteral feeds  -Hypothetical/knowledge MAEs  -Data for carer-caused MAEs are not separate from other data, e.g. from non-carer caused data or from other medication errors |
