# Supplementary material for: Carers' Medication Administration Errors in the Domiciliary Setting: A Systematic Review
Source: PLoS One. 2016 Dec 1;11(12):e0167204. doi: 10.1371/journal.pone.0167204 (PMC5132322; doi:10.1371/journal.pone.0167204)
Supplement: S5 Table — (DOCX) [file pone.0167204.s005.docx]

**S5 Table - Quality Assessment Scores**

| First author, Year [Country/Language] | Quality Assessment Scores |
| --- | --- |
| Absulem(2011) [US/English]([38](#_ENREF_38)) | 82.00 |
| Alander(2000) [US/English]([39](#_ENREF_39)) | 93.33 |
| Alomar et al (2011) [Saudi Arabia/English]([56](#_ENREF_56)) | 96.88 |
| Azkunaga et al (2013) [Spain/ Spanish]([57](#_ENREF_57)) | 90.00 |
| Cohen et al (2008) [US/English]([40](#_ENREF_40)) | 100.00 |
| Conroy et al[2003] [UK/English]([58](#_ENREF_58)) | 71.88 |
| Costa et al [2011] [US/English]([41](#_ENREF_41)) | 61.54 |
| Donelan et al [2002] [US/English]([25](#_ENREF_25)) | 75.00 |
| Goldman & Scolnik [2004] [Canada/English]([60](#_ENREF_60)) | 86.67 |
| Gribetz & Cronley [1987] [US/English]([42](#_ENREF_42)) | 93.75 |
| Guberman [1990] [Israel/Hebrew]([61](#_ENREF_61)) | 46.67 |
| Heubi et al (1998) [US/English]([43](#_ENREF_43)) | 63.33 |
| Hyam et al (1989) [Israel/English]([62](#_ENREF_62)) | 86.67 |
| Jonville et al (1991) [France/French]([66](#_ENREF_66)) | 87.50 |
| Kaushal et al (2007) [US/English]([44](#_ENREF_44)) | 100.00 |
| Lemer et al (2009) [US/English]([45](#_ENREF_45)) | 100.00 |
| Li et al (2000) [US/English]([46](#_ENREF_46)) | 90.63 |
| Lifshitz & Gavrilov (2000) [Israel/English]([63](#_ENREF_63)) | 80.00 |
| Llewellyn et al (2003) [Australia/English]([67](#_ENREF_67)) | 73.68 |
| Mattar et al (1975) [US/English]([47](#_ENREF_47)) | 50.00 |
| McErlean et al (2001) [US/English]([48](#_ENREF_48)) | 90.63 |
| McMahon et al (1997) [US/English]([49](#_ENREF_49)) | 81.58 |
| Moretti et al (2013) [France/French]([64](#_ENREF_64)) | 85.71 |
| Ni et al (2013) [China/Mandarin]([68](#_ENREF_68)) | 69.23 |
| Pelissier-delour et al (2007) [France/French]([65](#_ENREF_65)) | 90.63 |
| Ranelli & Hansen (1997) [US/English]([50](#_ENREF_50)) | 90.00 |
| Rivera-penera et al (1997) [US/English]([51](#_ENREF_51)) | 84.38 |
| Ryu & Lee (2012) [South Korea/English]([69](#_ENREF_69)) | 96.88 |
| Schaefer et al (2008) [US/English]([52](#_ENREF_52)) | 89.29 |
| Slatter et al ( 2004) [UK/English]([59](#_ENREF_59)) | 75.00 |
| Taylor J. et al (2006) [US/English]([53](#_ENREF_53)) | 100.00 |
| Taylor D (2009) [Australia/English]([31](#_ENREF_31)) | 100.00 |
| Travis et al (2000) [US/English]([54](#_ENREF_54)) | 90.00 |
| Walsh et al (2011) [US/English]([30](#_ENREF_30)) | 100.00 |
| Walsh et al (2013) [US/English]([29](#_ENREF_29)) | 100.00 |
| Zandieh et al (2008) [US/English]([55](#_ENREF_55)) | 96.88 |
